# Metastasis-associated protein 3 in colorectal cancer determines tumor recurrence and prognosis

**DOI:** 10.18632/oncotarget.16332

**Published:** 2017-03-17

**Authors:** Yi Huang, Yunlong Li, Fenfei He, Shiqi Wang, Yaohui Li, Gang Ji, Xiaonan Liu, Qingchuan Zhao, Jipeng Li

**Affiliations:** ^1^ State key Laboratory of Cancer Biology, National Clinical Research Center for Digestive Diseases and Xijing Hospital of Digestive Diseases, Fourth Military Medical University, Xi’an, China; ^2^ Department of Anesthesiology, Xijing Hospital, Fourth Military Medical University. Xi’an, China

**Keywords:** MTA3, colorectal cancer, immunohistochemistry, recurrence, prognosis

## Abstract

Metastasis-associated protein family (MTA) promotes tumor cell invasion and metastasis of human malignancies. However, the novel component of MTA family, MTA3 was found to play conflicting roles in human malignancies. While the expression pattern and potential function of MTA3 in colorectal cancer has not been addressed yet. In the present study, we investigated the protein expression of MTA3 by immunohistochemistry assay, analyzed its association with tumor progression, recurrence and prognosis in239 cases of patients. Results showed that MTA3 expression in colorectal cancer was significantly decreased in colorectal cancer compared with normal specimens. Its expression was found to be correlated with tumor differentiation, metastases and TNM stage. Kaplan–Meier analysis proved that MTA3 was associated with both disease-free survival and overall survival of patients with colorectal cancer that patients with negative MTA3 expression tend to have unfavorable outcome. Moreover, cox's proportional hazards analysis showed that negative MTA3 expression was an independent prognostic marker of poor outcome. These results provided the first evidence that MTA3 level was decreased in colorectal cancer and significantly correlated with tumor cell invasion and metastasis. It also demonstrated that MTA3 might serve as a potential marker of tumor recurrence and prognosis of colorectal cancer.

## INTRODUCTION

The MTA (Metastasis-Associated Protein) family, including MTA1, MTA2 and MTA3, are Mi-2/nucleosome remodeling and deacetylase (NuRD) protein complex [1]. Due to their ability of promoting or suppressing gene expression by changing chromatin structure, MTA family members are correlated with tumor carcinogenesis and metastasis [1]. However, the exact roles of the three MTA members in human malignancies might be diverse. It was found that MTA1 expression level was significantly increased in a variety of solid tumor, such as hepatocellular cancer, esophageal cancer, pancreatic cancer and breast cancer, and could facilitate tumor invasion and metastasis in these malignancies [2–5]. Compared with MTA1, MTA2 was reported to have high homology consistence in protein structure. In addition, a series of work showed that MTA1 could promote tumor cell invasion and metastasis in non-small cell lung cancer, colon cancer, gastric cancer and breast cancer [6–10]. These results indicated that MTA1 and MTA2 could play oncogenic roles in human malignancies and facilitate tumor cell invasion and metastasis. Nevertheless, in opposite to MTA1 and MTA2, MTA3 was reported to have different expression pattern and function in human malignancies, which was first identified in human breast cancer [11, 12]. The expression level of MTA3 was found to be decreased in human malignancies including ovarian cancer, breast cancer and endometrial cancer. Moreover, MTA3 was found to could inhibit tumor cell invasion and metastasis in these malignancies [13–15]. These opposing results indicated that MTA3 might play different roles in human malignancies compared with MTA1 and MTA2, which further suggested a wide range of function of MTA family. In human colorectal cancer, previous work revealed that MTA1 and MTA2 expression was up-regulated and correlated to tumor aggressiveness [7, 16–18]. However, till now, the expression level and potential function of MTA3 remains unclear in colorectal cancer.

Therefore, in order to clarify the expression level and explore the potential function of MTA3 in colorectal cancer, we investigated the protein expression level of MTA3 in clinical specimens by immunohistochemistry assay, analyzed the association of MTA3 level with clinicopathological characteristics and postoperative survival of patients in the present study.

## RESULTS

### MTA3 expression detected by immunohistochemical assay

**Figure 1:**
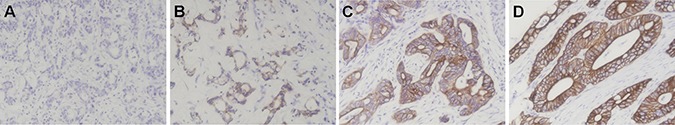
MTA3 staining in colorectal cancer detected by immunohistochemistry assay (200×) (**A**) negative staining of MTA3; (**B**) weak positive staining of MTA3; (**C**) moderate ositive staining of MTA3; (**D**) strong positive staining of MTA3.

In our study, MTA3 staining detected by immunohistochemistry assay was found mainly located in cytoplasm (Figure 1). According to the immunohistochemical staining evaluation protocol described in methods, negative MTA3 staining was detected in 178 colorectal cancer specimens. While 61 cases were defined as positive MTA3 staining in the 239 cases of colorectal cancer. Among the 56 cases of normal control specimens, negative MTA3 staining was detected in 14 cases of specimens and positive staining was found in 41cases of specimens. Based on staining classification, results of statistical analysis showed that the negative rate of MTA3 in colorectal cancer specimens was significantly increased, compared with that in normal control specimens (*P* < 0.05). These results indicated that protein expression level of MTA3 was down-regulated in colorectal cancer.

### Association of MTA3 level with clinicopathologic characteristics

As results showed a decreased expression pattern of MTA3, we further investigated the association of MTA3 level in colorectal cancer with clinicopathologic characteristics of patients involved. Statistical analysis results showed that MTA3 expression level in colorectal cancer was significantly correlated to tumor differentiation, node metastasis, distant metastasis as well as TNM stage, since negative MTA3 staining was more likely to be detected in tumors with poor differentiation (*P* < 0.001), node metastasis (*P* < 0.001), distant metastases (*P* = 0.034) or advanced TNM stage (*P* < 0.001). These results indicated that MTA3 might play a tumor suppressor role in differentiation and aggression in colorectal cancer. However, MTA3 expression level was not found to be associated with sex (*P* = 0.545), age at diagnosis (*P* = 0.596), tumor site (*P* = 0.890), tumor size (*P* = 0.204) or depth of invasion (*P* = 0.284). Results were showed in Table 1.

**Table 1:** Association of MTA3 expression with clinical features

Variable	*n*	MTA3 expression		*P*
		Negative	Positive	
Total	239	178	61	
**Sex**				0.545*
Male	141	103	38	
Female	98	75	23	
**Age at diagnosis**				0.596*
≤ 60	144	109	35	
> 60	95	69	26	
**Tumor site**				
Left colon	68	52	16	0.890*
Right colon	78	58	20	
Rectum	93	68	25	
**Tumor size**				0.204*
≤ 3.0 cm	82	57	25	
> 3.0 cm	157	121	36	
**Differentiation status**				< 0.001*
Well	41	17	24	
Moderately	103	75	28	
Poor	95	86	9	
**Depth of invasion**				0.284*
T_1_+ T_2_	92	65	27	
T_3_+ T_4_	147	113	34	
**Lymph node metastasis**				< 0.001*
Absent	92	57	35	
Present	147	121	26	
**Distant metastasis**				0.034*
Absent	208	151	57	
Present	31	28	3	
**TNM stage**				< 0.001*
I+ II	92	57	35	
III+ IV	147	115	26	

* *P* value when expression levels were compared using Pearson χ^2^ test

### Association of MTA3 level with disease-free survival

**Figure 2:**
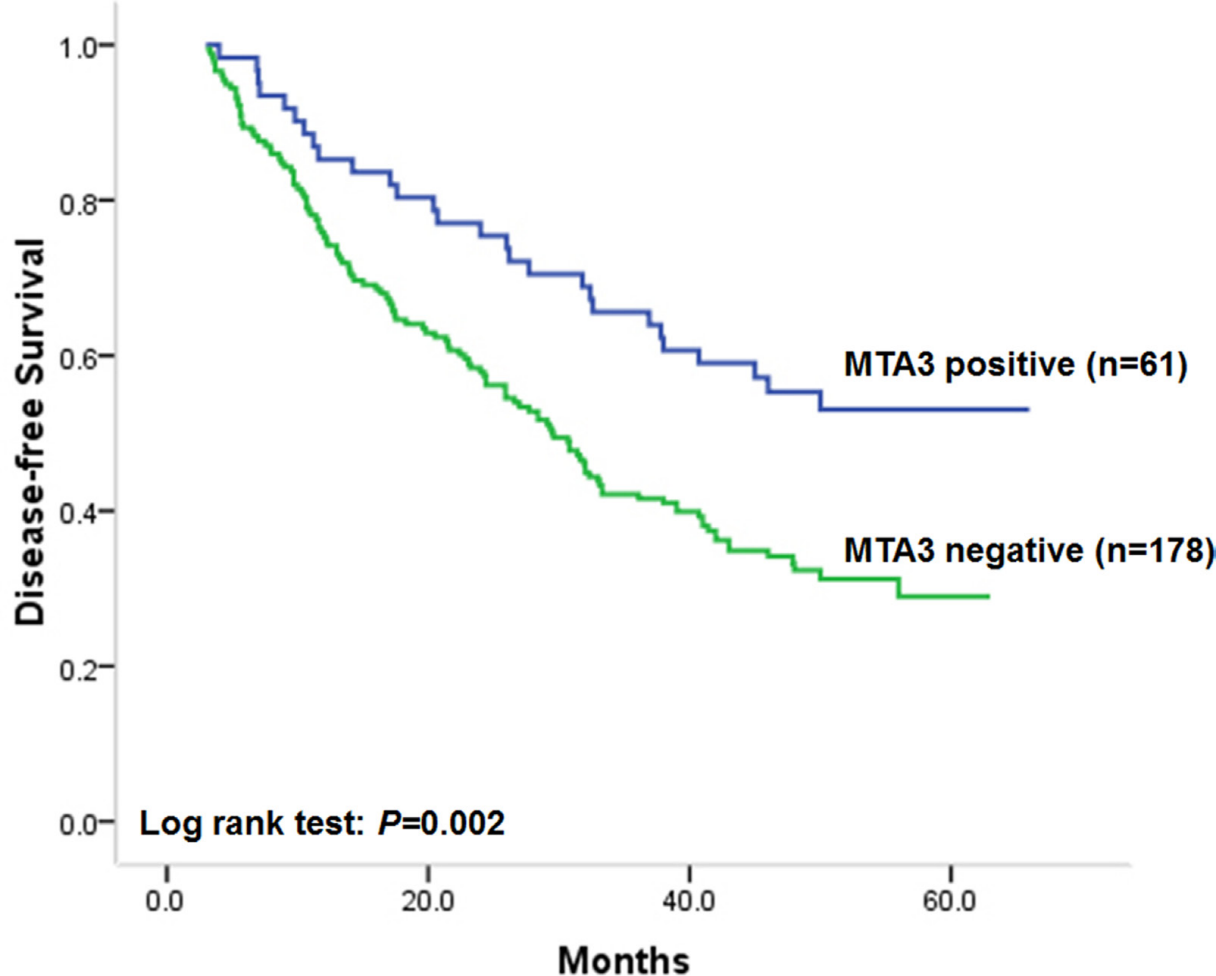
Kaplan–Meier postoperative survival analysis on disease-free survival for patients with colorectal cancer and MTA3 staining

Due to statistical analysis revealed a significant association between MTA3 level and tumor aggressiveness, we next investigated the association of MTA3 level in colorectal cancer with disease-free survival which depended on tumor invasion and metastasis. Results of Kaplan–Meier analysis proved that, compared with patients with MTA3 positive tumors, those with MTA3 negative tumors had unfavorable disease-free survival (Figure 2, log-rank test: *P* = 0.002). The median disease-free survival of patients with MTA3 negative tumors was 29.5 months (95% CI: 24.5–34.5). While the median disease-free survival time of patients with MTA3 positive tumors cannot be estimated due to more than half of patients survived. These results indicated that patients with tumors of positive MTA3 staining had lower risk of tumor recurrence. In addition, differentiation status (log-rank test: *P* = 0.012), invasion depth (log-rank test: *P* = 0.005), node metastasis (log-rank test: *P* = 0.002) and TNM stage (log-rank test: *P* < 0.001) were also found to be correlated to disease-free survival in univariate survival analysis, indicating that patients with tumors of poor differentiation, deep invasion, node metastasis or advanced TNM stage had unfavorable disease-free survival and higher risk of tumor recurrence. While no statistically significant correlations were found between disease-free survival and sex, age, tumor location or tumor size. Statistical results and unadjusted hazard ratio (HR) were showed in Table 2.

**Table 2:** Association of MTA3 and clinical factors with disease-free survival

	Unadjusted HR (95% CI)	*P*	Adjusted HR (95% CI)	*P*
MTA3 negative	1.91 (1.26–2.88)	0.002	1.78 (1.18–2.70)	0.006
Sex	0.91 (0.60–1.38)	0.907	0.93 (0.59–1.47)	0.753
Age at diagnosis	1.18 (0.85–1.64)	0.312	1.13 (0.81–1.56)	0.473
Tumor site	1.22 (0.81–1.86)	0.342	1.24 (0.83–1.95)	0.356
Tumor size	1.49 (0.95–2.36)	0.083	1.30 (0.59–2.85)	0.520
Differentiation status	1.70 (1.07–2.69)	0.026	1.64 (1.06–2.54)	0.035
TNM stage	5.76 (3.12–10.65)	< 0.001	4.20 (1.72–10.24)	0.002

* Hazard ratios in univariate models.

^†^ Hazard ratios in multivariable models.

Abbreviations: HR, hazard ratio; 95% CI, 95% confidence interval.

To verify the independent effect of MTA3 level on disease-free survival of patients involved, cox proportional hazards model analysis was performed adjusting for sex, age at diagnosis and tumor differentiation status, which aimed to control for confounding factors. Results showed that low MTA3 level was independently correlated to unfavorable disease-free survival of patients. The adjusted HR of patients with colorectal cancer of negative MTA3 staining was 1.78 (95% CI: 1.18–2.70 *P* = 0.006), compared with those with MTA3 positive tumors (Table 2). The results above suggested that patients with MTA3 negative colorectal cancer would have higher risk of tumor relapse than those with MTA3 positive ones.

### Association of MTA3 expression with overall survival

**Figure 3:**
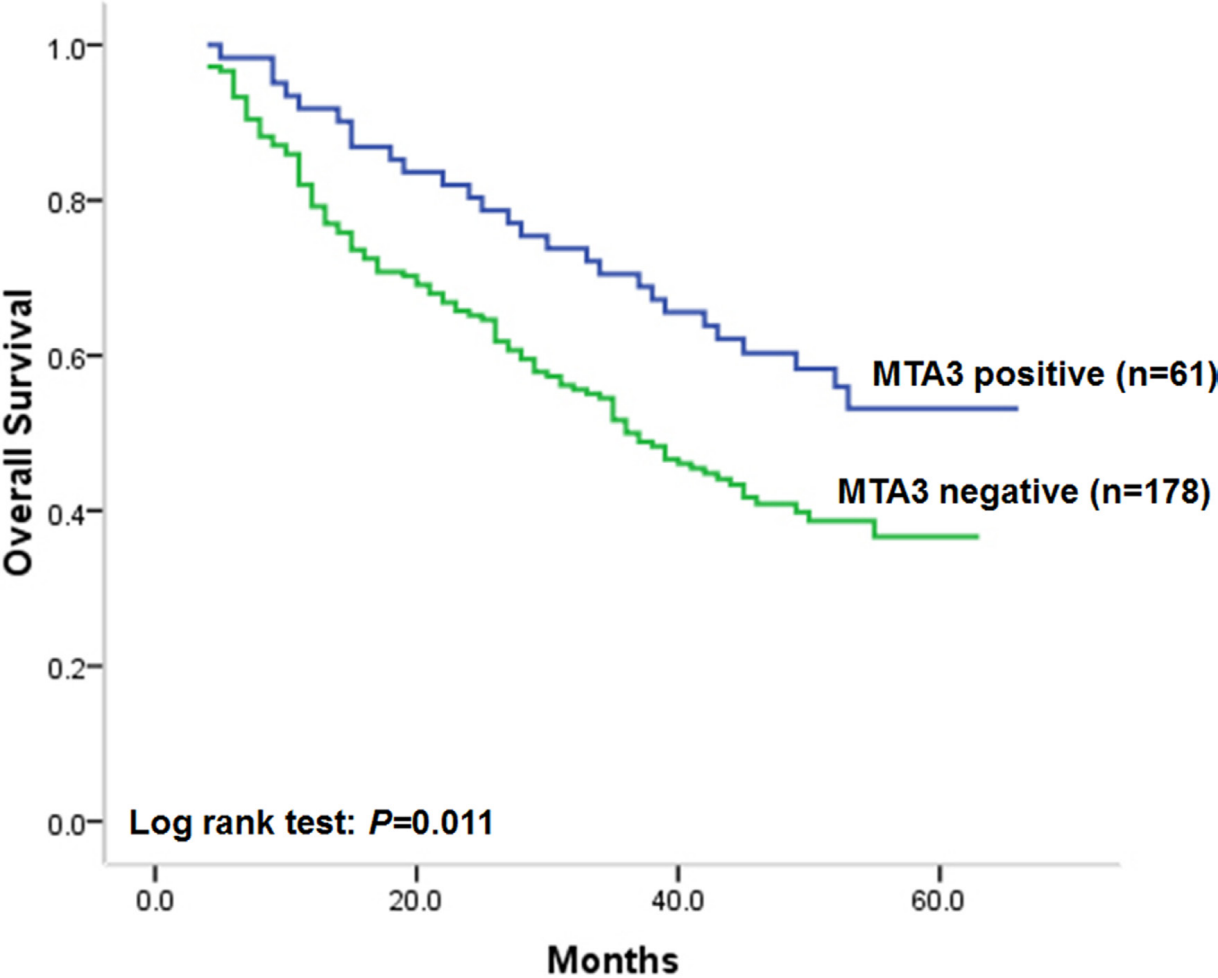
Kaplan–Meier postoperative survival analysis on overall survival for patients with colorectal cancer and MTA3 staining

Since univariate and multivariate analysis demonstrated that patients with MTA3 negative tumors had higher risk of tumor recurrence, we next carried out statistical analysis to investigate the association of MTA3 level in colorectal cancer with overall survival of patients. Similar to the results on disease-free survival, MTA3 level was found to be significantly correlated to overall survival of patients. Univariate survival analysis results showed that patients with MTA3 negative tumors had unfavorable overall survival, compared with those with MTA3 positive ones (Figure 3, log-rank test: *P* = 0.011). The postoperative median overall survival of patients with MTA3 negative tumors was 36.0 months (95% CI: 30.0–42.4). While the median overall survival time of patients with MTA3 positive tumors cannot be estimated due to more than half of patients survived. In addition, it was also found that overall survival of patients with colorectal cancer was closely correlated to differentiation status (log-rank test: *P* = 0.005), depth of invasion (log-rank test: *P* = 0.010), node metastasis (log-rank test: *P* = 0.001) and TNM stage (log-rank test: *P* < 0.001), indicating that patients with tumor of poor differentiation, deep invasion, node metastasis or advanced TNM stage had unfavorable overall survival. While no statistically significant correlations were found between overall survival of patients and sex, age, tumor location or tumor size and. Statistical results and unadjusted HR were showed in Table 3.

**Table 3:** Association of MTA3 and clinical factors with overall survival

	Unadjusted HR* (95% CI)	*P*	Adjusted HR^†^ (95% CI)	*P*
MTA3 negative	1.72 (1.12–2.62)	0.013	1.60 (1.04–2.44)	0.032
Sex	0.79 (0.52–1.21)	0.280	0.85 (0.54–1.36)	0.501
Age at diagnosis	1.18 (0.84–1.66)	0.347	1.17(0.82–1.67)	0.389
Tumor site	1.25 (0.71–2.22)	0.440	1.18 (0.63–2.20)	0.601
Tumor size	1.76 (0.86–3.06)	0.121	1.73 (0.82–2.96)	0.144
Differentiation status	2.24 (1.33–3.76)	0.002	2.16 (1.28–3.61)	0.005
TNM stage	6.43 (3.39–12.18)	< 0.001	4.56 (1.82–11.40)	0.003

* Hazard ratios in univariate models

^†^ Hazard ratios in multivariable models

Abbreviations: HR, hazard ratio; 95% CI, 95% confidence interval.

In multivariate cox proportional hazards analysis, results showed that MTA3 level in colorectal cancer could be an independent prognostic factor on overall survival after adjusting for sex, age at diagnosis, differentiation status and TNM stage, since low MTA3 level was independently correlated to unfavorable overall survival after controlling for all these factors. The adjusted HR of patients with MTA3 negative colorectal cancer was 1.60 (95% CI: 1.04–2.44 *P* = 0.032, Table 3), compared with those with MTA3 positive tumors (Table 3). Moreover, lymph node metastasis and TNM stage was also found to be independently correlated to overall survival of patients in multivariate analysis (Table 3).

## DISCUSSION

Colorectal cancer is one of the most common human malignancies all over the world [19, 20]. In this century, the incidence rate of colorectal cancer in China has been continually increasing, ranking it the third out of all cancer-related deaths [21]. Recently, molecules involved in cancer progression have been thought to could serve as markers for prognosis. Thus, better understanding of the pathogenesis and identification of novel biomarkers for colorectal cancer will shed light on the understanding of the molecular mechanisms underlying cancer progression, providing more effective management strategies. Recently, the members of MTA family were identified to be closely correlated to tumor aggressiveness. Till now, investigations on MTA3 have only limited to a few types of human malignancies. Moreover, limited publications indicated that MTA3 might play conflicting roles compared with the other two MTA members [22]. The expression level of MTA3 was found to be down-regulated in human brain glioma and closely correlated to clinicopathologic characteristics and outcome of patients [23]. While in human brain glioma cell lines, knockdown of MTA2 expression was found to could significantly suppress tumor cell growth, migration and invasion [24]. These results suggested that different MTA members might have diverse function in human malignancies.

Therefore, we conducted the present study to determine MTA3 expression level and its association with clinicopathologic characteristics, disease-free survival and overall survival of patients with colorectal cancer. Our study included a prospective hospital-based study cohort with large sample size and intimate information on outcome and clinicopathological characteristics. Results based on MTA3 staining classification proved that MTA3 level in colorectal cancer was down-regulated compared with that in normal tissues. Statistical analysis revealed that low MTA3 expression level in colorectal cancer was significantly associated with poor differentiation, node metastasis, distant metastases and advanced TNM stage. In colorectal cancer, negative MTA3 staining was more likely to be detected in tumors with poor differentiation, node metastasis, distant metastases or advanced TNM stage in our investigation. These results indicated that MTA3 might inhibit tumor differentiation and progression in colorectal cancer. As invasion and metastasis could determine tumor recurrence and corresponding outcome, we next analyzed the association of MTA3 level with disease-free and overall survival. In our study cohort, it was proved that low MTA3 level in colorectal cancer was correlated to unfavorable disease-free and overall survival. In not only univariate but also multivariate survival analysis, the prognostic impact of MTA3 for disease-free and overall survival was both statistically significant. These results indicated that MTA3 might be an independent molecular marker of tumor recurrence and survival for patients with colorectal cancer.

In MTA family, MTA3 is the latest identified member. Thus, the potential mechanism of MTA3 is still uncertain. It has been reported that MTA3 could inhibit the transcription of Snail gene and alter downstream gene expression including E-Cadherin, by which MTA3 may take part in the regulation of cancer cell invasion, metastasis and mediate epithelial to mesenchymal transition (EMT) [25, 26]. In breast cancer, MTA3 was demonstrated to could enhance cell-cell adhesion and inhibit tumor cell invasion and metastasis by its inhibition on Snail and E-cadherin [27, 28]. In hormone-independent types of cancer, MTA3 was also found to could suppress tumor cell invasion and metastasis by inhibiting Wnt-target genes through directly targeting the transcription of Wnt [29, 30]. These findings provided clues for the mechanism of the tumor suppressor role of MTA3 in colorectal cancer demonstrated by our investigation. However, further studies are still needed to explore the molecular mechanism of MTA3 in colorectal cancer.

In the present study, we demonstrated that MTA3 expression level in human colorectal cancer was down-regulated and significantly associated with tumor differentiation, invasion and metastasis. Our study also proved for the first time that MTA3 was independently correlated to disease-free and overall survival of patients with colorectal cancer. These results indicated that MTA3 might act as a tumor suppressor and potential prognostic marker in colorectal cancer

## MATERIALS AND METHODS

### Patients and specimens

The present research has been approved by the Ethics Committee of Fourth Military Medical University. The study cohort consisted of 239 cases clinical specimens from patients who were diagnosed as colorectal cancer between January 2008 and June 2009 in Xijing Hospital of Digestive Diseases has been validated in our previous publication [31]. The histomorphology of all tissue specimens were confirmed by the Department of Pathology, Xijing Hospital. Patients with following criteria were subsequently excluded: received treatment prior to surgery including neoadjuvant chemotherapy; harvested insufficient specimens for protein expression evaluation; diagnosed as colorectal stromal tumor; diagnosed with additional cancers; refused consent. Clinicopathologic information and follow-up data of the remaining 239 patients were prospectively entered into a database, which was under a close follow-up scheme and updated with respect to survival status every three month by telephone visit and questionnaire letters. Disease-free survival is defined as the time elapsed from surgery to the first occurrence of any of the following events: recurrence of colorectal cancer; colorectal cancer distant metastasis; development of second non-colorectal malignancy excluding basal cell carcinomas of the skin and carcinoma *in situ* of the cervix; or death from any cause without documentation of a cancer-related event. The diagnosis of recurrence and distant metastasis was based on the imaging method such as endoscope, ultrasonography, computed tomography, magnetic resonance imaging and position emission tomography, if possible, cytologic analysis or biopsy. Overall survival is defined as the time elapsed from surgery to death of patients with colorectal cancer. Death of participants was ascertained by reporting from the family and verified by review of public records. The disease-free and overall survival status was assigned by trained staff blinded to other clinicopathologic and exposure data.

### Immunohistochemistry assay and staining evaluation

Immunohistochemistry assay on MTA3 was performed in all the 239 cases of colorectal cancer and 56 cases of normal specimens. Fresh tissues were fixed in 10% formalin and embedded in paraffin wax. One of the deepest sections from each tumor was selected for evaluation, and 4-μm sections were examined by immunohistochemistry. Tissue sections were deparaffinized in xylene, and then rehydrated in graded concentrations of ethyl alcohol. Antigen retrieval was performed by placing the tissues in sodium citrate buffer and applying a high voltage for 3 min (pH 6.0), followed by natural cooling. Then sections were placed in 3% H_2_O_2_ for 10 min to inhibit the endogenous peroxide activity, washed three times with phosphate-buffered saline (PBS) buffer for 5 min and placed in normal goat serum as blocking antibody at room temperature for 10 min. The primary antibody against MTA3 utilized was rabbit polyclonal antibody (1:150; Proteintech, Chicago, IL, USA). After incubation at 4°C for 24 h, sections were washed three times with PBS buffer for 10 min. The samples were then incubated with the biotinylated goat anti-rabbit secondary antibodies at 37°C for 25 min. Subsequently, the sections were washed with distilled water and PBS for 3 min. Prepared DAB was dropped onto the slides, which were then incubated at 37°C for between 3 and 5 min. The sections were then counterstained in hematoxylin, washed with distilled water, differentiated with 1% hydrochloric acid alcohol, washed with distilled water. All sections were evaluated by pathologist without knowledge of the clinical information. MTA3 staining was classified by the following criteria: 0, negative; 1+, < 10% positive cells; 2+, 10–50% positive cells; 3+, > 50% positive cells. Specimens with 2+ or 3+ immunostaining were classified as positive MTA staining.

### Statistical analysis

Statistical analysis was carried out by the statistical package SPSS (version l3.0). Associations between MTA3 expression and categorical variables were analyzed by Pearson χ^2^ test. Correlation coefficients were analyzed by contingency or Spearman correlation analysis, as appropriate. Survival curves were estimated using the Kaplan–Meier method, and differences in survival distributions were evaluated by the log-rank test. Cox's proportional hazards modeling of factors potentially associated with survival was performed in order to identify which factors might have a significantly independent influence on survival. Differences with a *P* value of 0.05 or less were considered to be statistically significant.
